# Mitochondrial stress markers associate with phenotypic variability in Fabry disease

**DOI:** 10.1186/s13023-026-04461-9

**Published:** 2026-06-30

**Authors:** Lucia Lavalle, Hibba Kurdi, David Moreno Martinez, Vincent Muczynski, Simon Heales, Derralynn Hughes

**Affiliations:** 1 Cancer Institute, University College London, London, UK; 2 Lysosomal Storage Disorders Unit, Royal Free London NHS Foundation Trust, London, UK; 3 Institute of Cardiovascular Science, University College London, London, UK; 4 Neurometabolic Unit, National Hospital, Queen Square & UCL Great Ormond Street Institute of Child Health, London, UK

**Keywords:** Fabry disease, Mitochondrial unfolded protein response, Hsp60, Mitokines (FGF-21, GDF-15), Phenotypic heterogeneity

## Abstract

**Background:**

Fabry disease (FD) exhibits marked clinical heterogeneity that cannot be fully explained by residual α-galactosidase A activity. Mitochondrial dysfunction has been reported in FD, but the role of mitochondrial stress remains unexplored.

**Objective:**

To investigate whether mitochondrial unfolded protein response (mtUPR) related markers associate with phenotypic variability and correlates with disease severity.

**Methods:**

We measured intracellular heat-shock protein 60 (Hsp60) expression by western blotting in fibroblasts and peripheral blood mononuclear cells (PBMCs). In the clinical cohort, intracellular Hsp60 was measured in PBMC whole-cell lysates from 27 FD patients (14 males, 13 females). Serum fibroblast growth-factor-21 and growth differentiation-factor-15 were measured in 35 patients. Clinical outcomes included Mainz Severity Score Index, Age-Adjusting Severity Score, estimated glomerular filtration rate, and left-ventricular mass index (LVMI).

**Results:**

Hsp60 showed variability, with sex-specific associations. In males, higher Hsp60 correlated with lower LVMI (r^2^ = −0.82, *p* = 0.01) and preserved renal function in late-onset patients (r^2^ = 0.89, *p* = 0.006). In females, higher Hsp60 associated with higher LVMI (r^2^ = 0.66, *p* = 0.045) and greater clinical severity. Male patients had elevated growth differentiation-factor-15 vs controls (935 vs 559 pg/ml, *p* = 0.002). Both mitokines correlated with age and disease severity.

**Conclusions:**

mtUPR related markers exhibit sex- and genotype-specific patterns associated with disease severity, suggesting that mitochondrial stress contributes to phenotypic heterogeneity and support further longitudinal evaluation of Hsp60, FGF-21 and GDF-15 as candidate biomarkers of disease burden and treatment response.

**Supplementary information:**

The online version contains supplementary material available at https://doi.org/10.1186/s13023-026-04461-9.

## Introduction

Fabry disease (FD) is a progressive, X-linked lysosomal storage disorder caused by deficient α-galactosidase A activity, leading to globotriaosylceramide (Gb3) accumulation in multiple organ systems [1]. The clinical heterogeneity of FD is striking, ranging from severe classical phenotypes manifesting in childhood to late-onset variants with organ-specific manifestations that may not appear until middle age [2]. This phenotypic variability cannot be fully explained by residual enzyme activity alone, as patients with similar biochemical profiles often exhibit markedly different clinical courses [3]. Understanding the molecular mechanisms underlying this heterogeneity is crucial for optimising therapeutic strategies and improving patient outcomes.

Ageing and lysosomal storage disorders share common features of proteostatic stress, characterised by the accumulation of misfolded proteins and declining cellular quality control mechanisms [4, 5]. In Fabry disease, evidence suggests that cellular dysfunction extends beyond Gb3 accumulation, encompassing mitochondrial abnormalities, oxidative stress, and inflammatory responses [6, 7]. The progressive nature of FD manifestations with age suggests that cellular stress responses may become overwhelmed over time, contributing to organ dysfunction.

The mitochondrial unfolded protein response (mtUPR) represents a conserved quality control mechanism activated by mitochondrial proteostatic stress [8]. Upon detecting mitochondrial dysfunction, cells initiate a coordinated nuclear-mitochondrial signalling pathway that up-regulates protective factors, including mitochondrial chaperones such as heat shock protein 60 (Hsp60) [9]. Additionally, mitochondrial stress triggers the cellular release of signalling molecules termed mitokines, including fibroblast growth factor 21 (FGF-21) and growth differentiation factor 15 (GDF-15), which coordinate systemic metabolic responses [10, 11]. While engagement of mitochondrial stress responses is generally cytoprotective, sustained signalling may become maladaptive and contribute to pathology [12].

Several observations support a role for mitochondrial stress responses in the pathogenesis of Fabry disease. First, lysosomal dysfunction can impair mitochondrial function through disrupted calcium homeostasis and altered autophagy [13]. Second, the accumulation of misfolded α-galactosidase A variants may create additional proteostatic stress [14]. Third, the progressive, age-related nature of FD complications mirrors patterns seen in other conditions characterised by chronic mitochondrial stress signalling [15]. These effects may vary by variant class, as missense and nonsense GLA variants are associated with different molecular consequences [16].

We hypothesised that altered Hsp60 expression and mitokine levels are associated with phenotypic variability in Fabry disease and correlate with disease severity, particularly in cardiac and renal manifestations. Furthermore, we predicted that sex-specific differences in mitochondrial stress responses might help explain the different clinical patterns observed between male and female FD patients.

## Methods

### Study design and participants

This cross-sectional study received ethical approval from the Health Research Authority and Health and Care Research Wales (REC: 20/WM/0329) and the study was conducted in accordance with the Declaration of Helsinki. All participants provided written informed consent. The study included 36 patients with FD, all with confirmed GLA pathogenic variants and documented enzyme activity measurements at diagnosis, before Fabry-specific therapy was started. Intracellular Hsp60 was measured in PBMC whole-cell lysates from 27 patients (14 males, 13 females). Serum FGF-21 and GDF-15 were measured in 35 patients (18 males, 17 females). These analysis groups overlapped but were not identical, as PBMC and serum samples were not available for every participant. Most participants were already receiving Fabry-specific therapy at the time of sampling. Treatment data were extracted from clinical records, including treatment status at sampling, treatment type, enzyme replacement therapy (ERT) preparation where applicable, pharmacological chaperone therapy (PCT), age at treatment initiation and age at sampling. Treatment exposure was considered descriptively and included in exploratory analyses. Healthy controls for Hsp60 analyses included (*n* = 4; 3 males, 1 female). Serum mitokine analyses included a larger cohort of age- and sex-matched volunteers without known genetic disorders (*n* = 25; 17 males, 8 females).

### Clinical assessments

Clinical data were collected retrospectively from medical records. Disease severity was assessed using the Mainz Severity Score Index (MSSI) and Age-Adjusting Severity Score (AASS) [17]. The cardiac evaluation included electrocardiogram and cardiac magnetic resonance imaging, with left ventricular mass index (LVMI) calculated using the Devereux formula [18]. Left ventricular hypertrophy was defined as LVMI ≥ 78 g/m^2^ (males) or ≥74 g/m^2^ (females) [19]. Renal function was assessed by estimating glomerular filtration rate (eGFR) using the CKD-EPI equation [20]. Patients were classified as classical or late-onset based on residual enzyme activity and characteristic symptoms [21].

### Laboratory methods

#### Cell culture and protein analysis

Peripheral blood mononuclear cells (PBMCs) were isolated by density gradient centrifugation and cultured for 6 days in RPMI-1640 medium. Whole-cell lysates were prepared from primary cultured PBMCs and fibroblast cell lines using mammalian cell lysis buffer with protease inhibitors. Intracellular Hsp60 protein expression was measured by western blotting using an anti-Hsp60 monoclonal antibody (Bio-techne, NBP2-34670 H) with Na+/K+-ATPase as loading control. Samples were analysed across multiple gels. For female samples, a consistent control sample was included across all gels. For male samples, one of three wild-type control samples was included within each gel. Densitometric quantification was normalised within each gel to the loading control. Fibroblast cell lines GM00302, GM00881, GM00882 (Coriell Institute) were grown in EMEM medium.

#### Serum mitokine analysis

Based on manufacturer protocols, Serum FGF-21 and GDF-15 levels were measured using commercial ELISA kits (R&D Systems, DF2100 and DGD150).

### Statistical analysis

Data analysis was performed using GraphPad Prism 8.0. Continuous variables are presented as median with interquartile range. Group comparisons used Mann-Whitney U tests or Kruskal-Wallis tests with Dunn’s post-hoc correction. Correlations were assessed using Spearman’s rank correlation. Exploratory multiple linear regression was used to investigate predictors of mitochondrial stress markers and clinical outcomes. For models examining intracellular Hsp60, candidate predictors included GLA protein, GLA activity, age at sampling and lyso-Gb3, where available. For serum FGF-21 and GDF-15, Hsp60 was also included as a candidate predictor. For clinical outcome models, MSSI, AASS, LVMI and eGFR were assessed against Hsp60, FGF-21, GDF-15, GLA protein, GLA activity, age at sampling and lyso-Gb3, depending on data availability. Model size was restricted according to the number of available observations, particularly for LVMI and subgroup analyses. We applied Bonferroni correction for primary analyses (α = 0.01) to address multiple testing and reported effect sizes alongside *p*-values. Statistical significance was set at *p* < 0.05 for exploratory analyses.

## Results

### Patient characteristics

The clinical cohort included 36 patients with FD: 19 males and 17 females. Hsp60 analyses were performed in 27 patients, and serum mitokine analyses were performed in 35 patients, according to sample availability. Patient demographics are summarised in Table 1. Males with the N215S variant showed predominantly cardiac phenotypes, while non-N215S males exhibited more classical, multisystem disease. Female patients exhibited variable phenotypes, independent of their genotype.

**Table 1:** Patient demographics

Parameters	Males		Females
Overall	*N* = 19		*N* = 17
	N215S	non-N215S	
N (%)	9 (47.4)	10 (52.6)	
Age (years) at diagnosis			
Mean (SD)	48.9 (19.1)	24.5 (17.9)	37 (17.9)
Median (range)	54 (13 to 71)	26 (1 to 53)	37.5 (5 to 69)
Currently on treatment, n (%)	9 (100)	10 (100)	12 (70.6)
Enzyme replacement therapy, n (%)	1 (11.1)	6 (60)	6 (50)
Pharmacological chaperone therapy, n (%)	8 (88.9)	4 (40)	6 (50)
Age (years) at treatment commencement			
Mean (SD)	50.8 (18.7)	29.5 (18)	36.9 (15.7)
Median (range)	57 (15 to 71)	31.5 (2 to 53)	39 (12 to 63)
Classic Fabry disease cardinal features			
Acroparesthesias, n (%)	1 (11.1)	8 (80)	8 (47.1)
Fabry crisis, n (%)	0 (0)	4 (40)	4 (23.5)
Angiokeratoma, n (%)	1 (11.1)	3 (30)	3 (17.7)
Decreased sweating, n (%)	4 (44.4)	7 (70)	6 (35.3)
Cornea verticillata*, n (%)	0 (0)	2 (20)	2 (11.8)
Abnormal pure tone audiometry, n (missing)	4 (1)	6 (0)	11 (0)
Mean age (SD)	67 (6.4)	42.3 (6.9)	46.6 (21.7)
Median age (range)	65 (62 to 76)	41.5 (35 to 55)	57 (4 to 69)
LVH on image, n (%)	7 (77.8)	7 (70)	7 (41.2)
Mean age (SD)	57.4 (8.5)	42.2 (8.8)	54.7 (9.3)
Median age (range)	57 (45 to 71)	41 (29 to 53)	52 (42 - 69)
CKD stages (mL/min/1.73 m2)			
1 (>90)	4	4	8
2 (60 ± 89)	3	4	6
3A (45 ± 59)	1	1	3
3B (30 ± 44)	0	0	0
4 (>15 ± 29)	1	0	0
5 (<15 or on dialysis)	0	1	0
GI symptoms during childhood	1 (11.1)	5 (50)	4 (30.8)
Serious clinical outcomes**, n (%)	6 (66.7)	5 (50)	6 (46.2)
Overall severity at sampling			
Mild (MSSI < 20), n (%)	4 (44.4)	3 (30)	10 (5.9)
Moderate (MSSI = 20 - 40), n (%)	3 (33.3)	5 (50)	6 (35.3)
Severe (MSSI > 40), n (%)	2 (22.2)	2 (20)	1 (5.9)
Age adjusting score			
Mean (SD)	−5.7 (6.8)	8.7 (9.5)	5.8 (10.1)
Median (range)	−6.9 (−15.8 to 6.1)	7 (−9.6 to 22.2)	5.1 (−14.3 to 24.6)

* Cornea verticillata was not assessed for all patients

** Serious clinical outcomes include myocardial infarction, stroke, transient ischaemic attack, implantation of pacemaker or of implantable cardiovascular defibrillator, the development of atrial fibrillation, and chronic kidney disease (CKD) 3A (GFR < 59 ml/min/1.73 m2).N: number, SD: standard deviation, LVH: left ventricular hypertrophy, GI: gastrointestinal

### Mitochondrial stress response in Fabry fibroblasts: intracellular Hsp60 and Hsp10 expression in Fabry fibroblast cell lines

**Fig. 1:**
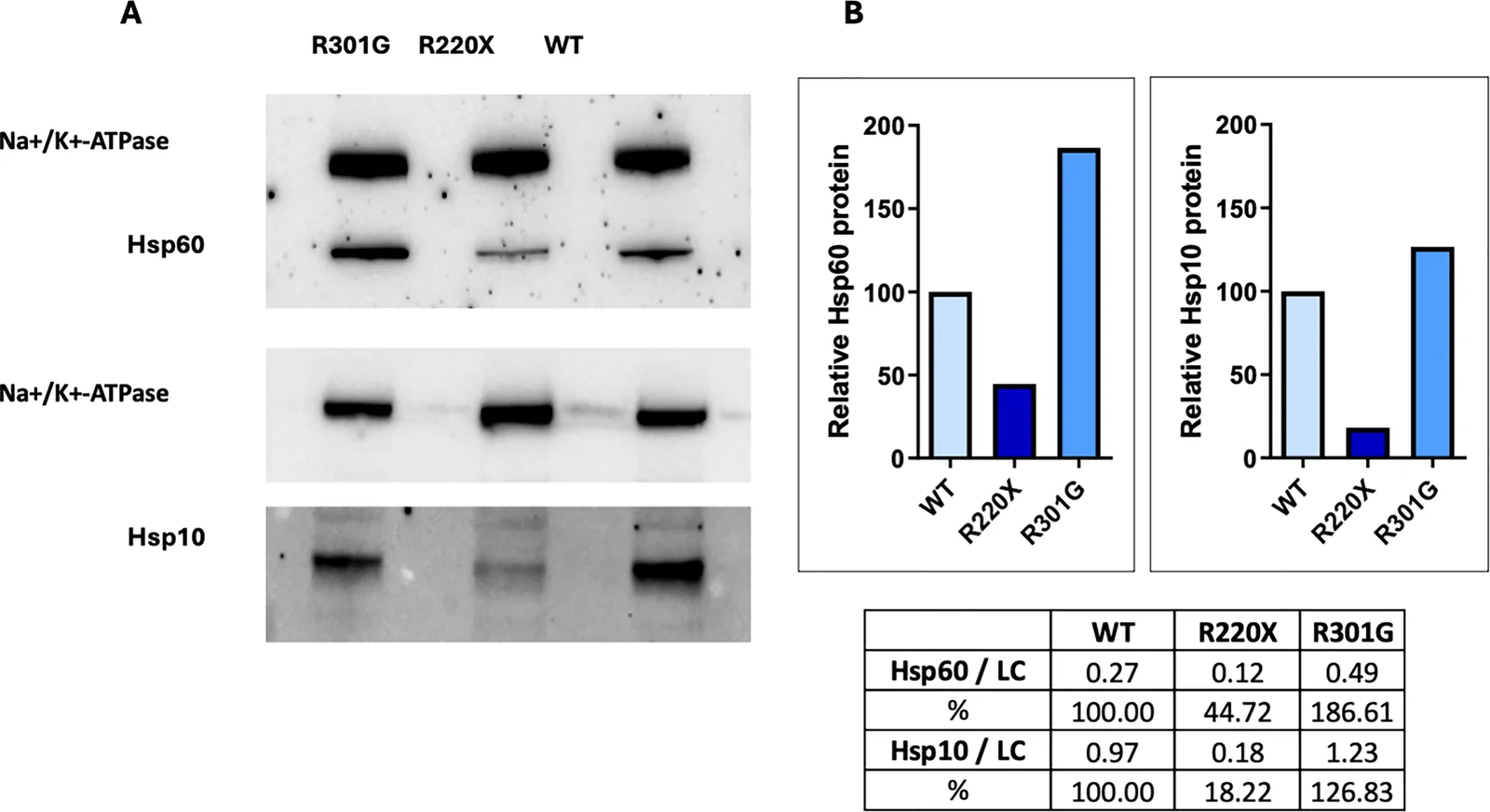
Genotype-specific heat shock protein expression in Fabry disease fibroblasts. (**A**) Representative western blots showing Hsp60 and Hsp10 expression in wild-type (WT), nonsense variant (R220X), and missense variant (R301Q) fibroblast cell lines. Na+/K+-ATPase serves as a loading control. (**B**) Quantification of heat shock protein levels normalised to loading control and expressed as a percentage of wild-type. The missense variant R301Q shows marked upregulation of both Hsp60 (187% of WT) and Hsp10 (127% of WT), while the nonsense variant R220X exhibits reduced expression (Hsp60: 45%, Hsp10: 18% of WT). Data represent single experiments from characterised cell lines. These findings suggest that missense variants causing protein misfolding trigger robust mitochondrial stress response than nonsense variants resulting in protein absence

Initial characterisation using immortalised fibroblast cell lines revealed fundamental differences in cellular stress responses between variant types (Fig. 1). The missense R301Q variant exhibited a marked elevation of both Hsp60 (187% of the wild-type level) and Hsp10 (127% of the wild-type level), consistent with mitochondrial stress responses involving the mtUPR pathway. In contrast, the nonsense R220X variant exhibited substantially reduced levels (Hsp60: 45%, Hsp10: 18% of wild-type).

These findings suggest distinct pathogenic mechanisms: missense variants create a “protein misfolding burden” that triggers compensatory stress responses. In contrast, nonsense variants result in “protein absence” without the additional proteostatic stress of misfolded protein accumulation. This distinction has implications for understanding why patients with similar residual enzyme activities can exhibit different clinical trajectories and treatment responses. Since the missense fibroblast line carried R301Q, these findings should be interpreted as supporting a variant-class model, not as direct evidence for all individual missense variants.

### Intracellular Hsp60 expression in Fabry disease patients

**Fig. 2:**
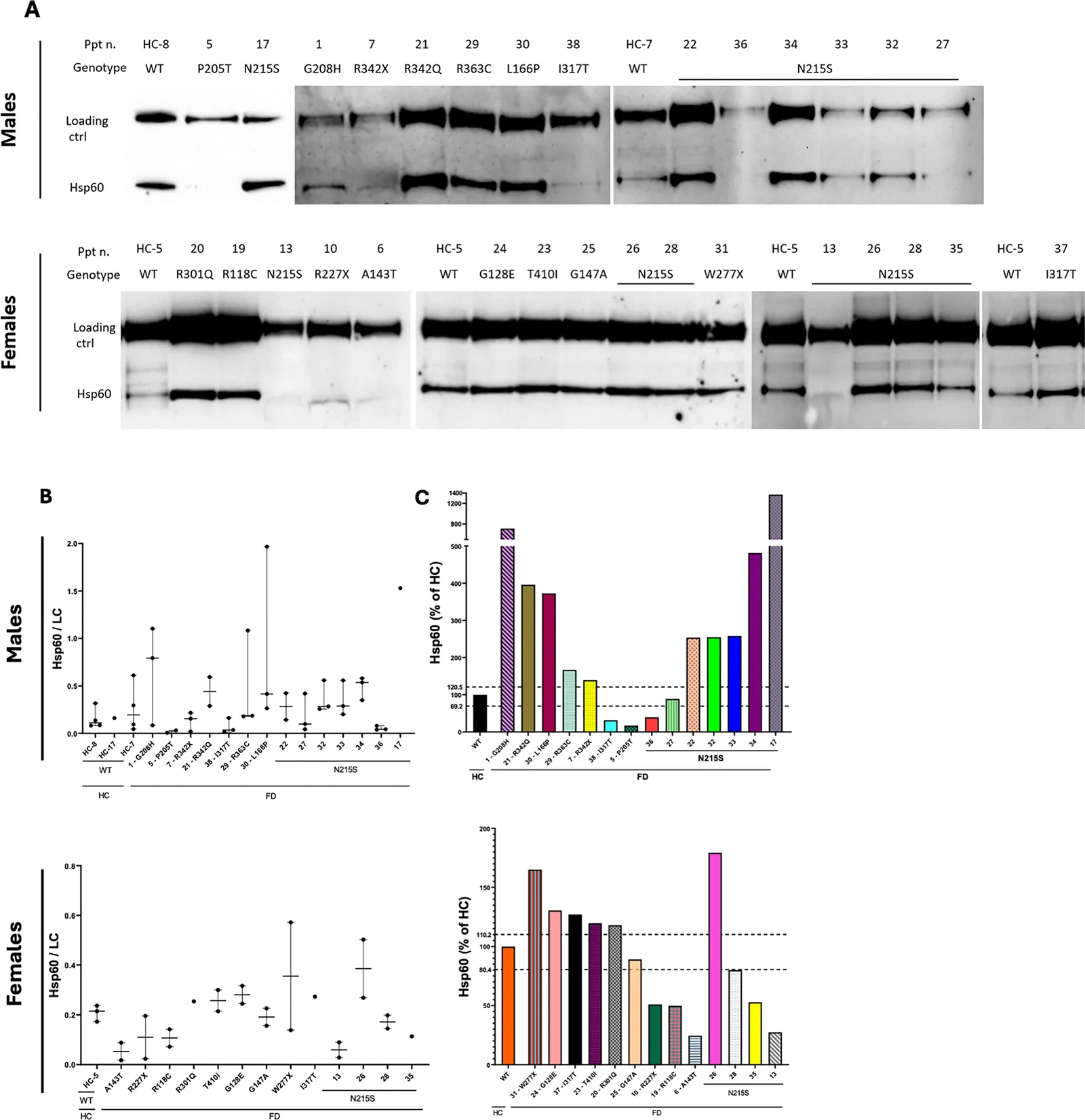
Intracellular Hsp60 protein expression levels in peripheral blood mononuclear cells from Fabry disease patients. (**A**) Representative western blots showing Hsp60 expression in PBMCs from healthy controls (HC) and Fabry disease (FD) patients with different genotypes. Na+/K+-ATPase serves as a loading control. (**B**) Quantification of Hsp60 levels for individual patients, normalised to Na+/K+-ATPase within each gel. Samples were analysed across multiple gels as described in the methods section. (**C**) Summary data showing Hsp60 levels as a percentage of healthy control median for males and females separately. While no significant overall difference exists between FD patients and controls, substantial inter-patient variability is observed, with some patients showing > 2-fold elevation and others < 50% of control levels. Males: *n* = 14 FD patients, *n* = 3 controls; Females: *n* = 13 FD patients, *n* = 1 control. Statistical analysis by Mann-Whitney U test; ns = not significant

Western blot analysis of PBMCs revealed substantial variability in intracellular Hsp60 protein levels among FD patients, with some individuals showing > 2-fold elevation and others < 50% of healthy control levels (Fig. 2). No significant overall difference was observed between FD patients and controls when analysed as a group (males: 254% vs 100%, *p* = NS; females: 89% vs 100%, *p* = NS).

**Fig. 3:**
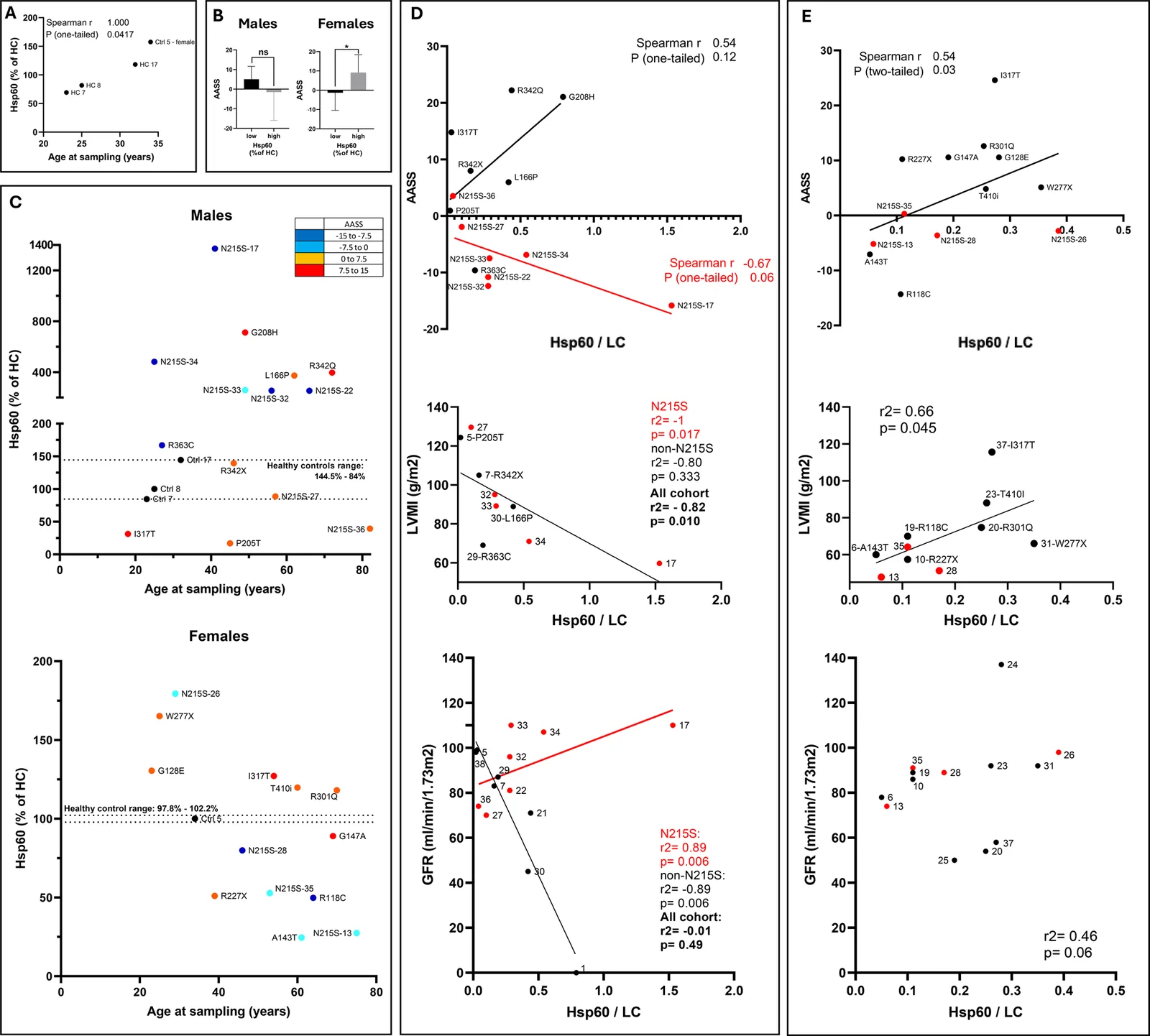
Associations between intracellular Hsp60 expression, age, clinical severity and organ-specific outcomes in Fabry. (**A**) Correlation between Hsp60 levels and age in healthy controls (*n* = 4) showing the expected positive relationship (r^2^ = 1.0, *p* = 0.04). (**B**) Age-adjusting severity Score (AASS) comparison between patients with high vs low Hsp60 levels, stratified by sex. In females, higher Hsp60 levels are associated with higher severity scores (*p < 0.05), while males show no significant association. (**C**) Correlation between Hsp60 levels and age in FD patients. In males, no significant association is observed. In females, a negative relationship is seen (r^2^ = −0.57, *p* = 0.02), in contrast to the positive age relationship observed in healthy controls. Data are coloured by AASS category. (**D**) Correlation analyses between intracellular Hsp60 levels (normalised to loading control) and clinical outcomes in male patients. Overall severity (AASS): separate analyses are shown for N215S patients (red symbols, negative trend: *r* = −0.67, *p* = 0.06) and non-N215S patients (black symbols, positive trend: *r* = 0.54, *p* = 0.12). Left ventricular mass index (LVMI): higher Hsp60 correlates with lower LVMI (r^2^ = −0.82, *p* = 0.01), with a particularly strong relationship in N215S patients (r^2^ = −1.0, *p* = 0.017). Glomerular filtration rate (GFR): N215S individuals show preserved renal function with higher Hsp60 (r^2^ = 0.89, *p* = 0.006), whereas non-N215S show the opposite pattern (r^2^ = −0.89, *p* = 0.006). (**E**) Correlation analyses between intracellular Hsp60 levels and clinical outcomes in female: higher Hsp60 levels correlate with greater AASS (r^2^ = 0.54, *p* = 0.03) and with higher LVMI (r^2^ = 0.66, *p* = 0.045). These findings indicate that altered mitochondrial stress responses in FD are independent of chronological ageing and exhibit sex- and genotype-specific associations with clinical severity and organ involvement

However, the relationship between Hsp60 and age differed markedly between patients and controls. While healthy controls showed the expected positive correlation between Hsp60 and age (r^2^ = 1.0, *p* = 0.04, Fig. 3A), this relationship was absent in FD patients (Fig. 3C), suggesting altered mitochondrial stress response independent of chronological ageing. Within the FD cohort, sex-stratified analysis showed no significant association in males and an inverse association in females (Fig. 3C).

### Sex-specific associations with clinical severity

Hsp60 levels showed sex-specific associations with disease severity. In females, higher Hsp60 levels correlated significantly with greater age-adjusted clinical severity (AASS: r^2^ = 0.54, *p* = 0.03, Fig. 3E) and with younger age at sampling (r^2^ = −0.57, *p* = 0.02, Fig. 3C), contrasting with the positive age correlation in healthy controls (Fig. 3A).

The relationship between Hsp60 and left ventricular hypertrophy revealed opposite patterns in males and females. In males, higher Hsp60 levels strongly correlated with lower LVMI (r^2^ = −0.82, *p* = 0.01), suggesting a cardioprotective effect (Fig. 3D). This relationship was most pronounced in the N215S subgroup. Conversely, in females, higher Hsp60 levels are associated with higher LVMI (r^2^ = 0.66, *p* = 0.045), indicating a potential maladaptive response (Fig. 3E). No significant association with lower eGFR was observed in females.

Association with renal function also differed by sex and genotype. In N215S males, again higher Hsp60 levels correlated with better preserved GFR (r^2^ = 0.89, *p* = 0.006), while non-N215S males showed the opposite relationship (r^2^ = −0.89, *p* = 0.006). Females showed a weak positive trend that did not reach statistical significance.

### Serum mitokine levels

**Fig. 4:**
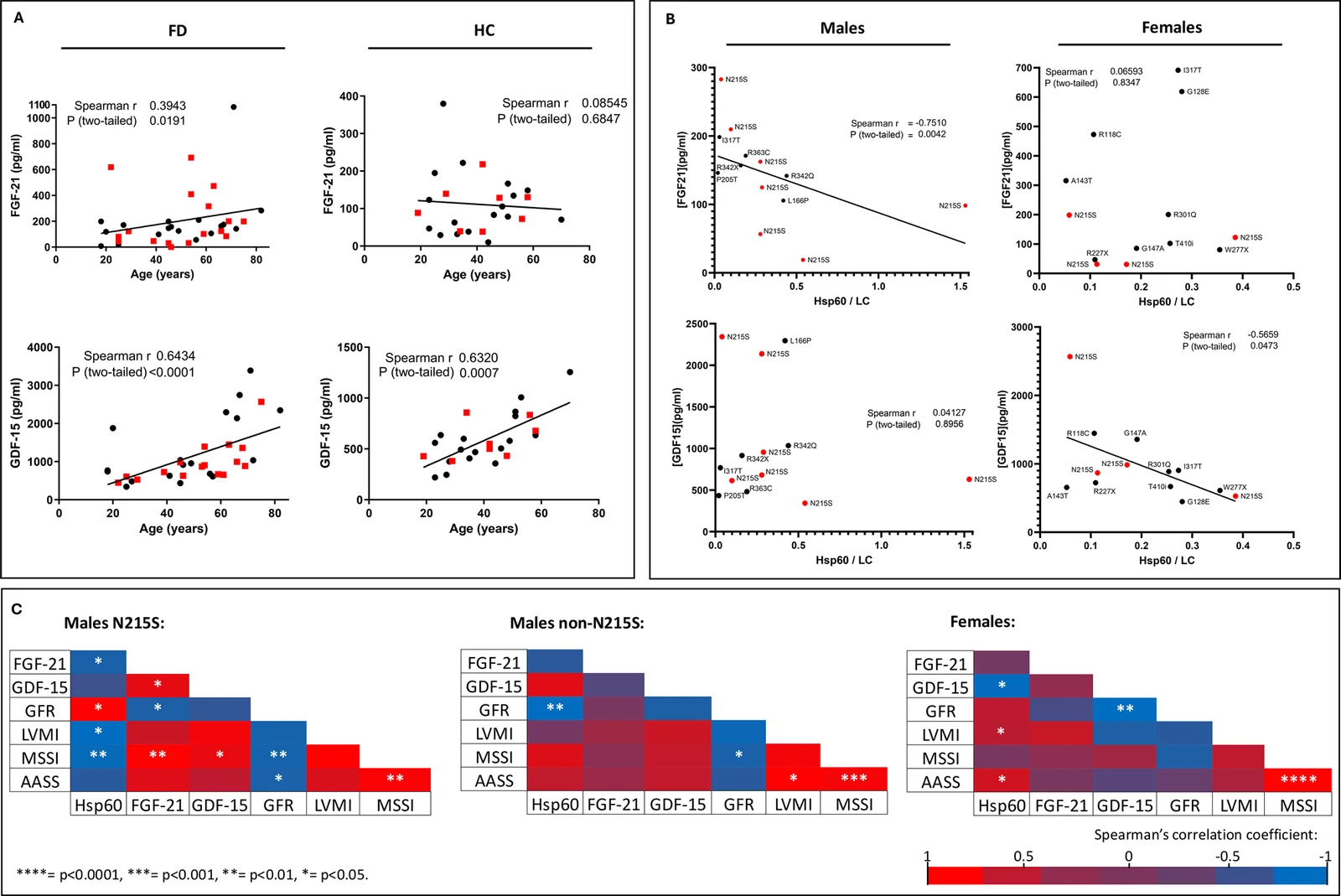
Relationships between circulating mitokines, ageing, intracellular Hsp60 and clinical outcomes in Fabry disease. (**A**) Correlation analyses between serum mitokines and age in Fabry disease (FD) and healthy controls (HC). FGF-21 shows a significant positive correlation with age in FD patients (*r* = 0.39, *p* = 0.02) but not in controls (*r* = 0.09, *p* = 0.68). GDF-15 demonstrates strong positive correlations with age in both FD patients (*r* = 0.64, *p* < 0.0001) and controls (*r* = 0.63, *p* = 0.0007), with a steeper slope in FD patients. Red symbols indicate female patients, and black symbols indicate male patients. These data suggest accelerated mitochondrial ageing in FD, with greater age-related increases in stress-responsive mitokines compared to healthy individuals. (**B**) Correlation analyses between intracellular Hsp60 levels and serum mitokines in FD patients. In males, higher Hsp60 correlates strongly with lower FGF-21 (*r* = −0.75, *p* = 0.004), while no significant correlation is observed in females. In females, higher Hsp60 correlates with lower GDF-15 (*r* = −0.57, *p* = 0.047), whereas no correlation is observed in males (*r* = 0.04, *p* = 0.90). N215S patients are indicated in red. (**C**) Summary correlation matrices showing the direction and strength of associations between intracellular Hsp60, serum mitokines, age, left ventricular mass index (LVMI), glomerular filtration rate (GFR), Mainz severity Score index (MSSI), and age-adjusting severity Score (AASS). These inverse and age-dependent relationships indicate coordinated but sex-specific mitochondrial stress responses, where intracellular stress-response activation may reduce the need for systemic mitokine signalling or reflect differential pathway activation across patient subgroups

Analysis of serum mitokines revealed significantly elevated GDF-15 levels in male FD patients compared to healthy controls (935 vs 559 pg/ml, *p* = 0.002). These levels also exceed published population medians for healthy males, including younger adults (<30 years: 483 pg/mL) and those aged 50–59 (931 pg/mL) [22] FGF-21 levels showed no significant difference (Figure S1). Serum FGF-21 and GDF-15 correlated positively with age in the FD cohort, with stronger correlations than observed in healthy controls (Fig. 4). Exploratory regression models suggested different patterns for the two mitokines: age was the main predictor of GDF-15 in males, N215S males and females, while FGF-21 was more closely related to Hsp60 and GLA protein in males. These findings suggest that the age-mitokine relationship is not uniform across mitokines and may be influenced by sex, genotype and clinical phenotype.

In males, Hsp60 levels showed a strong inverse correlation with FGF-21 (r^2^ = −0.75, *p* = 0.004), while females showed a weaker negative correlation with GDF-15 (r^2^ = −0.57, *p* = 0.047) (Fig. 4). These relationships suggest coordinated but sex-specific mitochondrial stress responses.

### Association with disease severity and outcomes

Both mitokines correlated positively with disease severity scores in males, with particularly strong associations in the N215S subgroup (MSSI vs FGF-21: r^2^ = 0.87, *p* = 0.005; MSSI vs GDF-15: r^2^ = 0.73, *p* = 0.03). No significant correlations were observed in females.

Patients with documented cardiomyopathy had significantly higher GDF-15 levels (935 vs 610 pg/ml, *p* = 0.02), and both mitokines were elevated in patients with left ventricular hypertrophy (FGF-21: 160 vs 98 pg/ml, *p* = 0.04; GDF-15: 975 vs 656 pg/ml, *p* = 0.01). Similar patterns were observed for clinically significant renal events, with GDF-15 showing the strongest associations (Fig. 4).

### Impact of treatment timing

Age at treatment initiation was associated with serum mitokine levels in exploratory analyses (Supplementary Figure S2). In males, later treatment initiation correlated with higher FGF-21 (r^2^ = 0.43, *p* = 0.04) and GDF-15 (r^2^ = 0.58, *p* = 0.006). In females, this association was observed for GDF-15 (r^2^ = 0.70, *p* = 0.007), but not clearly for FGF-21 or Hsp60.

In male patients, treatment-type stratified analyses showed different mitokine patterns (Supplementary Figure S3). Later pharmacological chaperone therapy initiation correlated with higher FGF-21 (r^2^ = 0.65, *p* = 0.002) and GDF-15 (r^2^ = 0.72, *p* = 0.008). In patients treated only with enzyme replacement therapy, later treatment initiation correlated with higher GDF-15 (r^2^ = 0.93, *p* = 0.003), but not FGF-21. These analyses were exploratory and may be influenced by age, genotype, phenotype severity and treatment indication.

### Predictive modelling

Multiple linear regression analysis identified key predictors of clinical outcomes. For MSSI in males, the best model (r^2^ = 0.76, *p* = 0.004) included GDF-15 (β = 0.01, *p* = 0.009), GLA protein levels (β = 37.3, *p* = 0.006), and Hsp60 (β = −52.2, *p* = 0.003), explaining 76% of severity score variance. In the N215S male subgroup, FGF-21 alone explained 92% of MSSI variance (β = 0.12, *p* = 0.01).

## Discussion

### Overall findings

This study provides the first systematic investigation of mtUPR related markers in Fabry disease, revealing complex sex- and genotype-specific patterns that may contribute to phenotypic heterogeneity. Hsp60 is induced as part of the mtUPR but is not specific to this pathway, while circulating mitokines reflect broader mitochondrial stress responses. Their coordinated alteration is consistent with engagement of mitochondrial stress pathways at both cellular and systemic levels [23, 24].

The most striking finding was the opposite relationship between intracellular PBMC Hsp60 protein expression levels and LVMI in males versus females. In males, higher Hsp60 levels were associated with better preserved cardiac architecture, while the relationship was reversed in females. This sex dimorphism may reflect fundamental differences in X-linked inheritance patterns, hormonal influences on mitochondrial function, and cellular stress responses.

### Sex-specific mitochondrial stress responses

The contrasting relationships between Hsp60 levels and LVMI in males versus females likely reflect X-linked inheritance patterns and hormonal influences on mitochondrial function [25]. In males who carry only one X chromosome, uniform GLA expression patterns may allow for more predictable mitochondrial stress responses. The protective association of higher Hsp60 with lower LVMI in males aligns with studies showing cardioprotective effects of moderate Hsp60 upregulation [26, 27].

Conversely, the positive correlation between Hsp60 and LVMI in females may reflect X-inactivation mosaicism leading to variable cellular stress responses [28]. In females, higher Hsp60 was seen in younger patients and was associated with higher AASS and LVMI. This pattern may indicate that in severely affected female patients, mitochondrial stress responses may become maladaptive in contexts of overwhelming cellular stress.

The observation that females showed a negative correlation between Hsp60 and age, contrasting with the positive correlation in healthy controls, suggests that normal ageing patterns are disrupted. This may be related to the modulatory effects of oestrogen on mitochondrial function and stress responses [29].

### Mitokine elevation and clinical implications

Elevated GDF-15 levels in male FD patients align with its established role as a stress-responsive mitokine in cardiovascular and renal disease [30]. GDF-15 has emerged as a biomarker of mitochondrial dysfunction and is elevated in various cardiovascular conditions [31]. In FD, Gregório et al. reported that GDF-15 was associated with cardiac and renal involvement in classical patients receiving enzyme replacement therapy, and also noted higher GDF-15 levels in patients who initiated ERT after 40 years of age [32].

Comparative biomarker studies suggest that GDF-15 provides a more global signal across mitochondrial disease phenotypes, while FGF-21 is more influenced by muscle involvement [33]. Consistent with this, mechanistic studies indicate that mitochondrial stress in skeletal muscle is a major driver of FGF-21 secretion, whereas GDF-15 integrates mitochondrial stress signals across tissues [34]. This distinction was also seen in our exploratory models: age was the main predictor of GDF-15, whereas FGF-21 showed stronger links with Hsp60 and GLA protein in males. Therefore, the age-related mitokine findings should be interpreted cautiously, as they may reflect a mixture of ageing, genotype and organ involvement.

The strong relationship between treatment timing and mitokine levels has important clinical implications. Patients initiating therapy after age 40 showed markedly elevated mitokine levels, suggesting a greater burden of mitochondrial stress at the time of intervention. This observation supports current discussions supporting early therapeutic intervention, even in asymptomatic patients [35]. Is late treatment just a surrogate for late onset patients?

### Genotype-specific responses: implications for disease mechanisms

The striking differences in mitochondrial stress marker patterns between N215S and non-N215S patients suggest distinct pathogenic mechanisms may contribute to these Fabry disease subtypes. These findings support the view that FD should not be considered a uniform lysosomal storage disorder and raise the possibility of genotype-specific therapeutic considerations.

#### Protein misfolding vs. Protein absence paradigms

The N215S variant exemplifies the later-onset missense mutation with cellular trafficking abnormalities within Fabry disease. This missense mutation results in a thermolabile enzyme that misfolds in the endoplasmic reticulum (ER), triggering ER-associated degradation (ERAD) and subsequently activating downstream stress responses [36]. Our fibroblast data demonstrate that missense variants, such as R301Q, produce robust Hsp60 upregulation (187% of wild-type), consistent with a pronounced mitochondrial stress response in response to proteostatic stress.

This misfolded protein burden creates a dual pathology: (1) enzyme deficiency leading to substrate accumulation and (2) continuous proteostatic stress from attempted protein folding and degradation. The ER-mitochondria contact sites (mitochondrial-associated membranes) serve as critical communication hubs where ER stress can contribute to mitochondrial stress signalling [37]. In N215S patients, this may create a chronic state of integrated stress response activation that may fundamentally alter cellular metabolism and stress tolerance.

Conversely, nonsense variants like R220X represent a “protein absence disorder,” where mRNA degradation via nonsense-mediated decay prevents the accumulation of misfolded proteins [38]. Our data show minimal Hsp60 upregulation (45% of wild-type) in these variants, suggesting that the absence of misfolded protein reduces proteostatic stress despite equivalent or greater substrate accumulation. This suggests the relevant pathology is primarily lysosomal dysfunction without the added burden of protein misfolding stress.

#### Therapeutic implications of mechanistic differences

These mechanistic distinctions have therapeutic implications. N215S patients showed that 92% of MSSI variance was explained by FGF-21 levels alone, indicating a tight coupling between mitochondrial stress and clinical severity. This suggests that therapies targeting proteostasis (such as pharmacological chaperones) may be particularly beneficial for missense variants by reducing the protein misfolding burden and consequently decreasing proteostatic and mitochondrial stress [39].

In addition, treatment-type stratified analyses in males showed different mitokine patterns. Later pharmacological chaperone therapy initiation was associated with higher FGF-21 and GDF-15, whereas later enzyme replacement therapy initiation was associated with higher GDF-15 only. These findings should not be interpreted as evidence of treatment superiority, but suggest that therapies with different mechanisms may have distinct relationships with mitochondrial stress markers.

For nonsense variants, the primary pathology stems from substrate accumulation rather than protein misfolding stress. These patients may benefit more from substrate reduction or enzyme replacement if their cellular stress responses are less overwhelmed by proteostatic burden [40].

#### Sex-specific interactions with genotype

The interaction between genotype and sex adds another layer of complexity. In N215S males, higher Hsp60 levels strongly correlated with better renal function (r^2^ = 0.89, *p* = 0.006), suggesting that a more effective mitochondrial stress response may be associated with cytoprotection when proteostatic stress is manageable. However, even high Hsp60 levels are associated with worse outcomes in females with severe phenotypes, possibly reflecting X-inactivation mosaicism creating cellular populations with varying stress tolerance [41].

This sex-genotype interaction may explain why some female N215S patients develop severe phenotypes despite this variant’s “late-onset” classification. Cellular mosaicism may create focal areas of high misfolded protein burden that overwhelm local stress responses, leading to tissue-specific pathology despite overall preserved enzyme activity.

These observations highlight the need for future work that clarifies the cellular basis of these sex- and genotype-specific responses, including how mitochondrial stress responses are modulated across different tissues.

#### Evolutionary and clinical perspectives

From an evolutionary perspective, preserving missense variants like N215S in the population, despite their pathogenic potential, may reflect residual protein function under optimal conditions. The amenability of many missense variants to pharmacological chaperone therapy supports this concept^3 6^. However, chronic proteostatic stress may accelerate cellular ageing processes, explaining the delayed but progressive nature of complications in these patients.

Clinically, these findings suggest that Fabry disease represents at least two distinct disorders: a “protein misfolding lysosomal disease” (exemplified by N215S and similar variants) and a “classical lysosomal storage disease” (exemplified by nonsense variants). This distinction may require different monitoring strategies, with mitokine levels being potentaillyuseful for the former group, and traditional biomarkers (lyso-Gb3, clinical symptoms) being more relevant for the latter.

The therapeutic window concept could also differs between groups. Missense variant patients may have a narrower therapeutic window due to cumulative proteostatic damage, explaining why early intervention is particularly relevant for preventing irreversible mitochondrial dysfunction. Nonsense variant patients may have more predictable progression patterns based primarily on substrate accumulation kinetics and so while early therapy is not less relevant it is more obviously prompted by the onset of clinical features.

### Therapeutic implications

These findings have several potential therapeutic implications. First, mitokine levels, particularly GDF-15, may serve as exploratory biomarkers for monitoring treatment response and disease progression. Second, the relationship between treatment initiation age and mitochondrial dysfunction markers supports further investigation of early treatment strategies. Third, sex-specific differences in mitochondrial stress responses may require tailored treatment approaches.

The observation that PCT showed different associations with mitokine levels compared to ERT suggests that treatment modalities targeting protein folding may have distinct effects on mitochondrial stress responses, although this requires confirmation in longitudinal treatment-stratified cohorts.

Mitochondrial stress markers may also help refine treatment stratification by identifying patients with greater vulnerability to mitochondrial dysfunction or differential responses to specific therapies

### Study limitations

Several limitations warrant consideration. The cross-sectional design precludes the determination of causality between mtUPR related markers and clinical outcomes. In addition, the markers used here do not provide definitive evidence of mtUPR activation.

The relatively small sample size, particularly for subgroup analyses, limits statistical power and generalisability. Given the modest numbers within sex- and genotype-stratified analyses, these findings should be regarded as exploratory and hypothesis-generating and may be susceptible to statistical artefact. The study design also does not allow full adjustment for potential confounders, including age, treatment status, genotype, co-morbidities, and other clinical variables.

The fibroblast experiments included one missense and one nonsense GLA variant. Since individual missense variants may differ in protein conformation, residual enzyme activity, trafficking, chaperone responsiveness and phenotype, these findings should be interpreted as supporting a variant-class model and not as representative of all missense variants. Studies including N215S and other common missense variants would be needed to test this directly.

We measured Hsp60 in PBMCs, which may not accurately reflect organ-specific mitochondrial stress. PBMCs also represent a heterogeneous cell population, and inter-individual variation in cellular composition may influence Hsp60 levels independently of disease-related stress responses. Tissue-specific markers would provide more direct evidence of organ-level mitochondrial stress. Furthermore, PBMCs were cultured before analysis, and ex vivo culture conditions may themselves influence stress protein expression. Another important limitation relates to Hsp60 quantification, which was performed by western blotting. This is a semi-quantitative technique and is subject to technical and inter-experimental variability despite normalisation to a loading control. Samples were analysed across multiple gels; a consistent reference control sample was used across gels for female experiments. However, in male experiments different control samples were used across gels, and inter-gel variability therefore cannot be fully excluded.

We cannot exclude the influence of co-morbidities, medications, or environmental factors on the mitochondrial stress markers assessed. Future studies should include a comprehensive assessment of potential confounders.

Finally, the mechanistic basis for sex-specific differences remains unclear. Functional studies examining patterns of X-inactivation, hormonal influences, and cellular stress responses are required to elucidate the underlying mechanisms.

## Conclusions

This study shows that mtUPR related markers exhibits sex- and genotype-specific patterns in Fabry disease that are associated with cardiac and renal disease severity. Higher intracellular Hsp60 levels are associated with lower LVMI in males but higher in females, while elevated serum mitokines, particularly GDF-15, are associated with disease severity and late treatment initiation.

These findings suggest that mitochondrial stress responses may contribute significantly to phenotypic heterogeneity in Fabry disease and may have potential as biomarkers for disease monitoring and optimisation of treatment timing. The strong association between treatment initiation age and mitokine levels supports early therapeutic intervention is consistent with greater mitochondrial stress in patients treated later in the disease course and suggests that mitochondrial dysfunction may become irreversible beyond a critical age threshold.

Future longitudinal studies are needed to validate these markers for clinical use and to elucidate the mechanistic basis for sex-specific differences in mitochondrial stress responses. Understanding these pathways may lead to new therapeutic targets and personalised treatment approaches for Fabry disease.

## Electronic supplementary material

Below is the link to the electronic supplementary material.

Supplementary Material 1

Supplementary Material 2

Supplementary Material 3

Supplementary Material 4

Supplementary Material 5

Supplementary Material 6

Supplementary Material 7

## Data Availability

The datasets generated and analysed during the current study are available from the corresponding author on reasonable request.
