# Supplementary material for: Mitochondrial stress markers associate with phenotypic variability in Fabry disease
Source: Orphanet J Rare Dis. 2026 Jun 30;21:326. doi: 10.1186/s13023-026-04461-9 (PMC13587467; doi:10.1186/s13023-026-04461-9)
Supplement: Supplementary file 1 [file 13023_2026_4461_MOESM2_ESM.pdf]

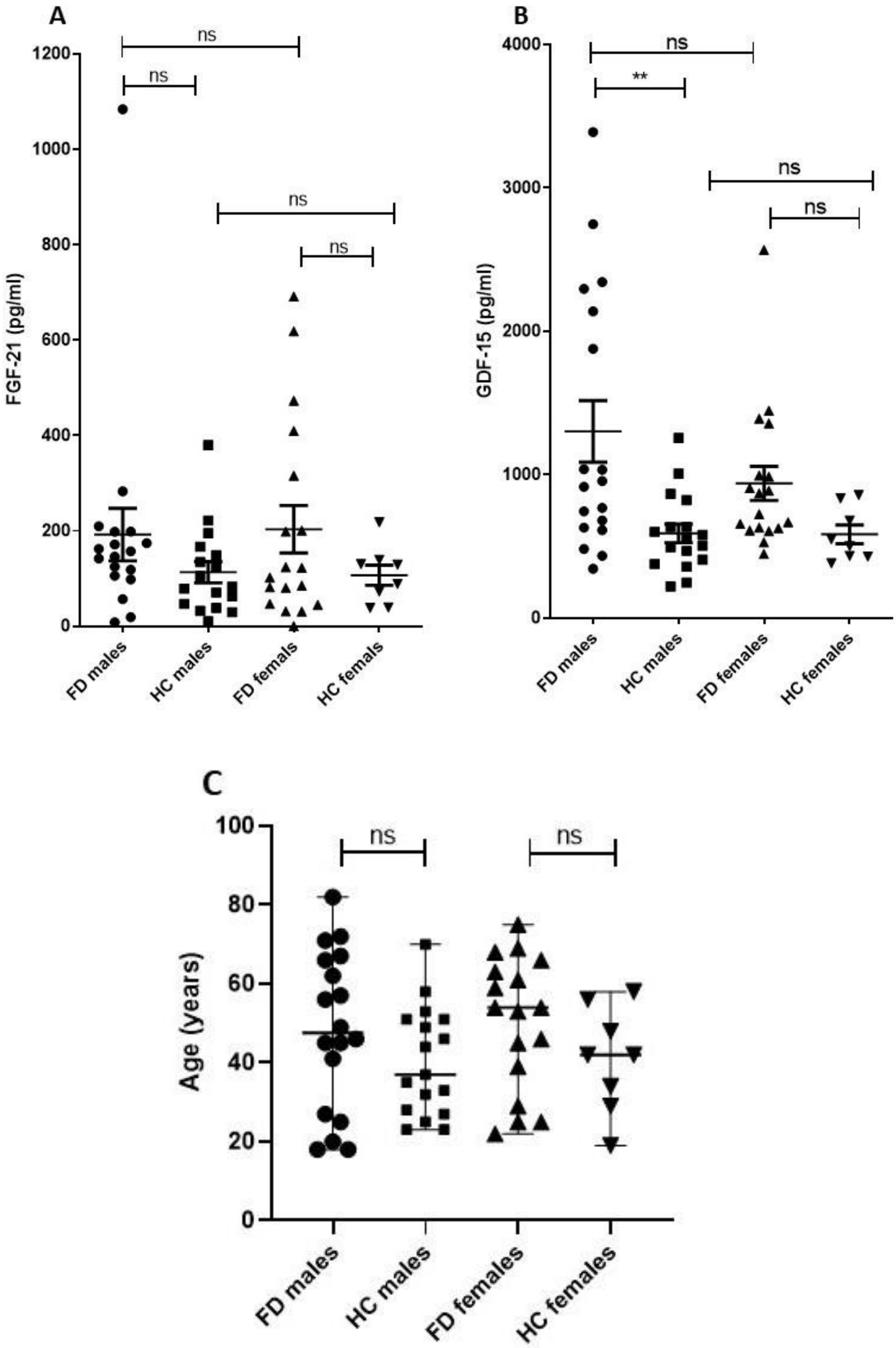

	Males		Females	
	FD	HC	FD	HC
Number of subjects	18	17	17	8
Age range (years)				
Mean (SD)	48.2 (20.2)	40.3 (13.8)	50.2 (17)	41 (13.3)
Median (range)	47.5 (18 - 82)	37 (23 - 70)	54 (22 - 75)	42 (19 - 58)
FGF21 (pg/ml)				
Mean (SD)	192.1 (232.6)	113.3 (91.8)	203.2 (210.8)	106.9 (60)
Median (range)	151.6 (8.1 - 1084)	83.1 (10.1 - 379.7)	112.4 (0.38 - 691.4)	108.7 (38.4 - 218.1)
GDF15 (pg/ml)				
Mean (SD)	1301 (914.6)	589.9 (270.7)	938.7 (504.5)	582.8 (186.1)
Median (range)	934.9 (342.6 - 3389)	559.3 (219.4 - 1255)	795.9 (447 - 2568)	525.4 (381.2 - 856.9)
