# Supplementary material for: Mitochondrial stress markers associate with phenotypic variability in Fabry disease
Source: Orphanet J Rare Dis. 2026 Jun 30;21:326. doi: 10.1186/s13023-026-04461-9 (PMC13587467; doi:10.1186/s13023-026-04461-9)
Supplement: Supplementary file 3 [file 13023_2026_4461_MOESM4_ESM.pdf]

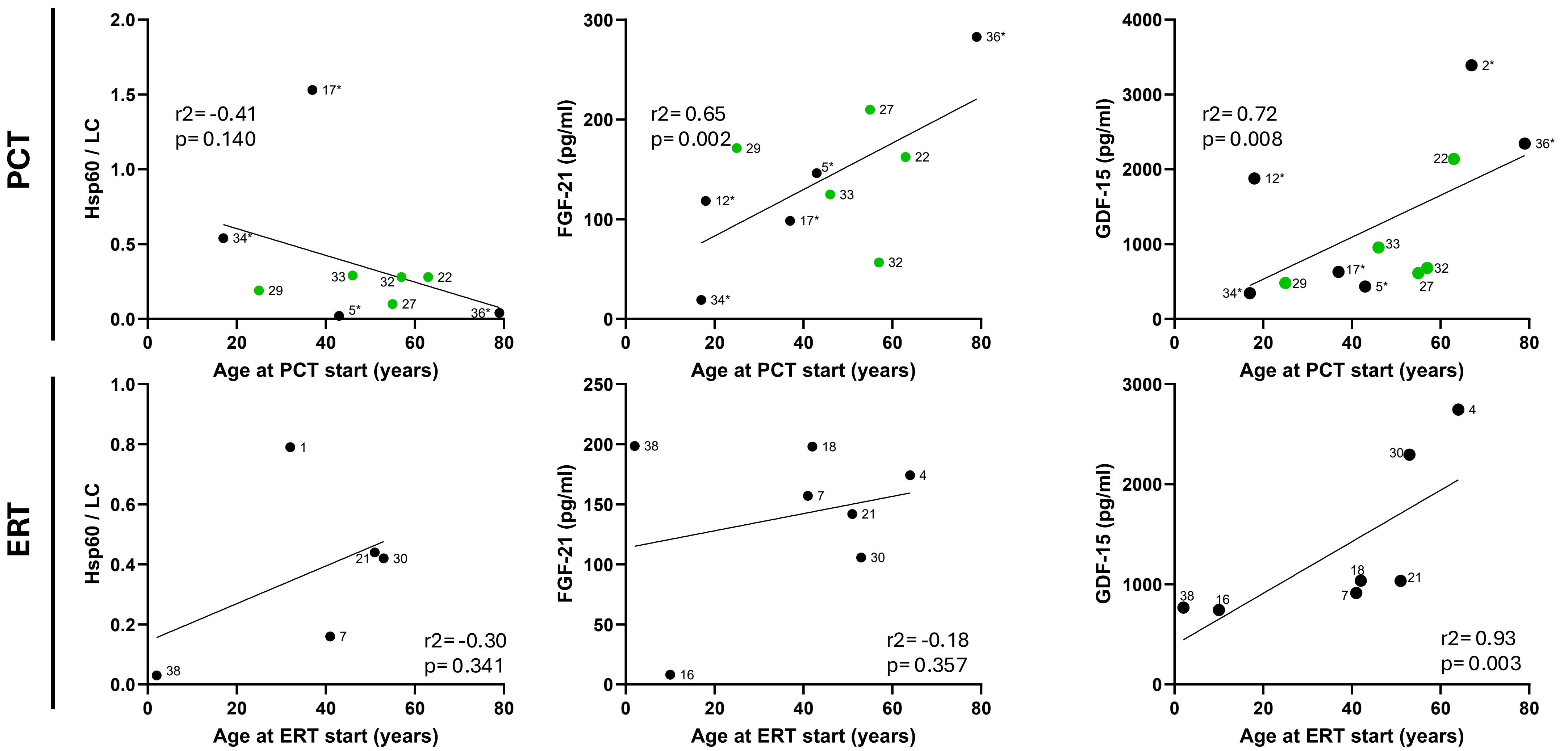
PCT
ERT
Hsp60 / LC
FGF-21 (pg/ml)
GDF-15 (pg/ml)
Age at PCT start (years)
Age at ERT start (years)
r2= -0.41
p= 0.140
r2= 0.65
p= 0.002
r2= 0.72
p= 0.008
r2= -0.30
p= 0.341
r2= -0.18
p= 0.357
r2= 0.93
p= 0.003
