# Supplementary material for: Mitochondrial stress markers associate with phenotypic variability in Fabry disease
Source: Orphanet J Rare Dis. 2026 Jun 30;21:326. doi: 10.1186/s13023-026-04461-9 (PMC13587467; doi:10.1186/s13023-026-04461-9)
Supplement: Supplementary file 4 [file 13023_2026_4461_MOESM5_ESM.pdf]

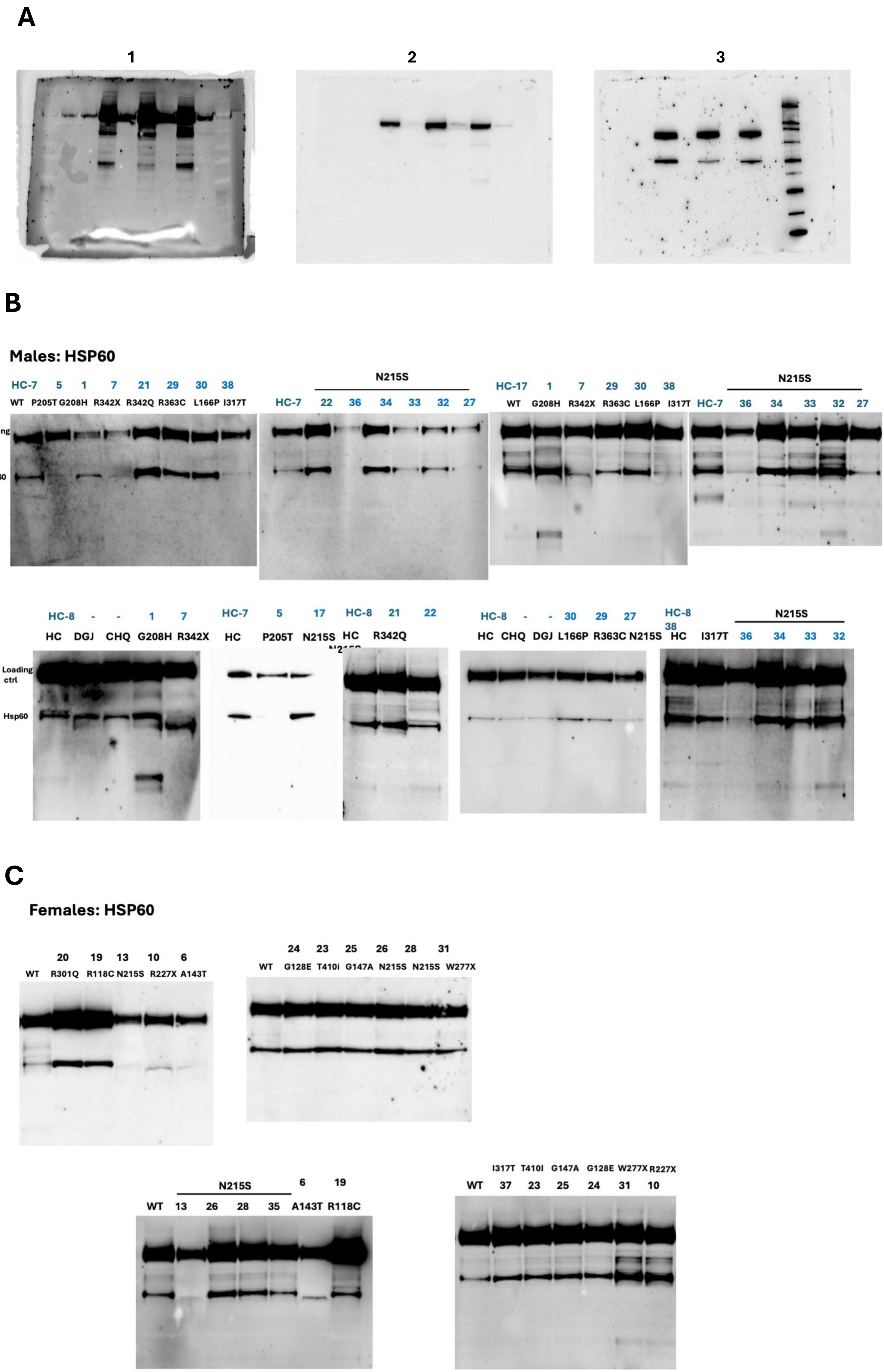

Supplementary file: uncropped western blots. (A) Fibroblasts experiments showing: 1. Hsp10 blot, 2. loading control (Na/K ATPase) blot, and 3. Blot for both, Hsp60 and loading control. **(B)** Full uncropped Hsp60 blots for male patients with corresponding Na/K ATPase loading controls. **(C)** Full uncropped Hsp60 blots for females with corresponding NA/K ATPase loading controls.
