# Supplementary material for: Mitochondrial stress markers associate with phenotypic variability in Fabry disease
Source: Orphanet J Rare Dis. 2026 Jun 30;21:326. doi: 10.1186/s13023-026-04461-9 (PMC13587467; doi:10.1186/s13023-026-04461-9)
Supplement: Supplementary file 5 [file 13023_2026_4461_MOESM7_ESM.pdf]

Supplementary Table 1. Individual treatment characteristics of Fabry disease participants, including treatment type, age at treatment initiation, age at sampling and treatment status at the time of sample collection.
