# Supplementary material for: Mitochondrial stress markers associate with phenotypic variability in Fabry disease
Source: Orphanet J Rare Dis. 2026 Jun 30;21:326. doi: 10.1186/s13023-026-04461-9 (PMC13587467; doi:10.1186/s13023-026-04461-9)
Supplement: Supplementary file 6 [file 13023_2026_4461_MOESM1_ESM.docx]

**Supplementary figure S1. Elevated serum mitokine levels in Fabry disease patients**. Serum levels of (A) fibroblast growth factor-21 (FGF-21) and (B) growth differentiation factor-15 (GDF-15) were measured by ELISA in FD patients and healthy controls stratified by sex. (C) Demographic characteristics of study participants. Male FD patients show significantly elevated GDF-15 levels compared to healthy male controls (935 vs. 559 pg/ml, **p=0.002), while FGF-21 levels and both mitokines in females show no significant differences from controls. Individual data points are shown with median and interquartile range. Statistical analysis by Mann-Whitney U test; **p<0.01, ns = not significant. These findings suggest sex-specific mitochondrial dysfunction patterns in FD.

**Supplementary figure S2. Impact of treatment on mtUPR markers.** Correlation analyses between markers of mtUPR and age at treatment commencement. Patients who started on enzyme replacement therapy (ERT) are in **black** whilst those who started on pharmacological chaperon therapy (PCT) are in **green**. Subjects with * started on ERT and switched to PCT.

**Supplementary figure S3. Impact of treatment type on mtUPR markers in male subjects with Fabry disease.** Correlation analyses between markers of mtUPR and age at treatment commencement. (A) Patients who are currently on pharmacological chaperon therapy (PCT) and (B) patients who have only been on enzyme replacement therapy (ERT). Patients who started on ERT are in black whilst those who started on PCT are in **green**. Subjects with * started on ERT and switched to PCT.
